# Development and validation of cardiorespiratory fitness prediction equations from 6-minute walk test: the Coronary Artery Risk Development in Adults (CARDIA) study

**DOI:** 10.1093/gerona/glag040

**Published:** 2026-02-14

**Authors:** Bjoern Hornikel, Erin E Dooley, Christopher Barrett Bowling, Baojiang Chen, Pablo Martinez-Amezcua, David R Jacobs, Mercedes Carnethon, Barbara Sternfeld, Cora E Lewis, Stephen Sidney, Priya Palta, Kelley P Gabriel

**Affiliations:** Department of Epidemiology, University of Alabama at Birmingham, Birmingham, Alabama, United States; Department of Epidemiology, University of Alabama at Birmingham, Birmingham, Alabama, United States; Durham Veterans Affairs Medical Center (VAMC), Durham Veterans Affairs Geriatric Research Education and Clinical Center, Durham, North Carolina, United States; Department of Medicine, Duke University, Durham, North Carolina, United States; Department of Biostatistics and Data Science, UTHealth Houston School of Public Health in Austin, Austin, Texas, United States; Department of Epidemiology, Johns Hopkins Bloomberg School of Public Health, Baltimore, Maryland, United States; Division of Epidemiology and Community Health, School of Public Health, University of Minnesota, Minneapolis, Minnesota, United States; Department of Preventive Medicine, Northwestern University, Chicago, Illinois, United States; Division of Research, Kaiser Permanente Northern California, Pleasanton, California, United States; Department of Epidemiology, University of Alabama at Birmingham, Birmingham, Alabama, United States; Division of Research, Kaiser Permanente Northern California, Pleasanton, California, United States; Department of Neurology, University of North Carolina at Chapel Hill, Chapel Hill, North Carolina, United States; Department of Epidemiology, University of Alabama at Birmingham, Birmingham, Alabama, United States

**Keywords:** Physical fitness, VO_2_peak, Cohort studies

## Abstract

**Background:**

Assessing maximal oxygen consumption (VO_2_max), the gold standard for assessing cardiorespiratory fitness, is often impractical in large-scale studies. We derived sex-specific VO_2_ max and graded exercise test duration (GXTd) prediction equations from 6-minute walk test (6MWT) performance.

**Methods:**

Data were from 564 Coronary Artery Risk Development in Young Adults (CARDIA) participants (mean age: 61.5 years; 58% women; 40% Black) who completed the 6MWT and symptom-limited modified Balke treadmill graded exercise test at the Year 35 (2021-2022) follow-up exam. Sex-stratified samples were randomly split (2/3 for training, 1/3 for testing) to derive and evaluate prediction equations. Stepwise linear regression identified predictors of VO_2_max and GXTd from 6MWT distance. Sex-specific VO_2_max CARDIA equations were compared with Burr and FRIEND equations. Models’ accuracies were evaluated by comparing the predicted values to measured values using Student’s *t*-test and Pearson correlation coefficients (*r*). Bland−Altman plots used to evaluate agreement between measured and predicted values.

**Results:**

Sex-specific VO_2_max CARDIA equations explained 53% and 57% of the variance in men and women, respectively, with strong correlations between measured and predicted values (*r* = 0.73 and 0.78). The Burr and FRIEND equations showed larger biases and weaker correlations compared with the CARDIA equations. The GXTd equations explained 59% and 62% of the variance in men and women, with strong correlations (*r* = 0.71 and 0.72) and no significant mean differences between observed and predicted.

**Conclusions:**

The CARDIA prediction equations for VO_2_max and GXTd from 6MWT enhance accuracy and accessibility, providing a practical tool for large-scale studies and clinical assessments, particularly in aging populations.

## Introduction

Cardiorespiratory fitness (CRF), the ability of the circulatory and respiratory systems to deliver oxygen to working muscles during physical activity and exercise, provides important diagnostic and prognostic information.^1^ Specifically, strong scientific evidence supports the inverse association between CRF and cardiovascular disease,^2^ all-cause mortality,^3^ and other adverse health outcomes such as obesity, type 2 diabetes, hypertension, and metabolic syndrome.^2^^,^^4^^,^^5^ These associations highlight the utility of CRF assessments for screening and risk stratification in adults.

The criterion measure of CRF is the direct measurement of maximal oxygen consumption (VO_2_max) during a maximal graded exercise test (GXT). However, this method is resource-intensive, requiring specialized equipment to capture gas exchange, trained personnel, and maximal exertion from participants, making the protocol less practical to implement in large-scale, population-based studies. To address these limitations, indirect measures of CRF, such as submaximal exercise tests and field-based protocols, have been developed to estimate VO_2_max.^6^ Among these alternatives, the 6-minute walk test (6MWT), a submaximal exercise test measuring the distance walked in 6 min, is easy to administer across a wide-range of expertise and skill levels, requires minimal equipment, is well-tolerated and safe across clinical and non-clinical populations, and has demonstrated its validity in CRF classification in comparison to the criterion in adults.^7^ Several studies have validated the utility of the 6MWT to predict VO_2_max; however, its generalizability is limited by small sample sizes, homogeneity in study populations (eg, predominantly White adults), or a focus on specific clinical populations.^8^-11 Further, research has shown that sex differences in fitness exist, with lower fitness levels in women compared to men due to differences in body composition, hemoglobin concentrations, and maximal cardiac output.^12^

Several cohort studies, such as the Coronary Artery Risk Development in Young Adults (CARDIA) study and Cooper Center Longitudinal Study, have relied on the duration of GXT (graded exercise test duration (GXTd); ie, time to exhaustion) to estimate CRF.^5^^,^^13^ GXTd has demonstrated strong predictive validity for VO_2_max in smaller laboratory-based studies of active and sedentary young adults (*r* ≥ 0.88),^14^^,^^15^ as well as within middle-aged to older adults in the population-based CARDIA cohort (*r* = 0.83).^16^ Predicting GXTd from 6MWT distance could provide an additional practical tool for CRF estimation, particularly in aging populations where maximal exertion tests are less feasible.

Developing sex-specific VO_2_max prediction equations using the 6MWT in a racially/ethnically diverse cohort could enhance their accuracy in predicting CRF in diverse populations. In addition, equations that estimate treadmill performance from the 6MWT can support continuity in CRF assessment over time, particularly when maximal testing becomes less feasible in aging or large-scale, population-based studies. Linking past treadmill assessments with current 6MWT performance preserves the integrity of CRF tracking over time and enhances our ability to study its impact on future health. This study aimed to develop sex-specific prediction equations for VO_2_max based on 6MWT distance within the CARDIA cohort, a diverse and well-characterized longitudinal prospective cohort study. It also sought to examine the accuracy of the newly developed equations in comparison to existing 6MWT and non-exercise VO_2_ prediction equations commonly used in clinical and research settings. Comparing these equations will allow us to evaluate whether the new CARDIA-derived equations offer improved accuracy and fit for a cohort of middle-aged to older adults relative to existing approaches. By addressing gaps in the literature, these newly developed equations could provide a practical, accessible, and non-invasive method for CRF assessment, particularly in aging populations or settings where traditional testing is impractical.

## Methods

CARDIA is a population-based longitudinal investigation of the development of cardiovascular risk factors and subclinical and clinical cardiovascular disease. Beginning in 1985-1986, 5115 Black and White young adults (18-30 years of age) were enrolled for an in-person examination at one of four clinical centers across the United States (Birmingham, Alabama, United States; Chicago, Illinois, United States; Minneapolis, Minnesota, United States; and Oakland, California, United States), with 10 additional in-person examinations held approximately every 2-5 years,^17^ to date. This study includes a subgroup of participants who attended the Year 35 (Y35; 2021-2022) in-person follow-up examination and consented to additional measures including a symptom-limited treadmill GXT and 6MWT, as part of two separate ancillary studies (CARDIA ACT-HF and CARDIA Function, respectively). All CARDIA participants provided written consent. Study procedures for the Y35 core exam and all ancillary studies were approved by the University of Alabama at Birmingham (UAB) Institutional Review Board (IRB); all other Field Centers agreed to rely on the UAB IRB as the single IRB for CARDIA.

### Participant descriptives

Standardized questionnaires and protocols were used to collect participant characteristics, such as age, sex assigned at birth (male or female), self-identified race (Black or White), education [high school (or equivalent) degree or less; or associate’s degree or more], smoking status (current, former, never), and meeting physical activity guidelines (≥300 exercise units as assessed by the self-reported CARDIA Physical Activity History questionnaire, which estimates a minimum weekly frequency and duration).^18^ Height was measured to the nearest 0.5 cm using a stadiometer and weight to the nearest 0.5 lb (0.2 kg) using a calibrated balance-beam scale, both taken in light clothing without shoes. Body mass index (BMI) was calculated using measured weight in kilograms and height in meters (kg/m^2^) and BMI categories were defined as healthy weight (≤24.9 kg/m^2^), overweight (25.0-29.9 kg/m^2^), and having obesity (≥30 kg/m^2^).

### Graded exercise treadmill testing

Medical eligibility for the GXT was determined during a screening using the American College of Sports Medicine criteria.^19^ Participants were ineligible to participate in the GXT if they had a history of heart, blood vessel, or lung conditions, severe hypertension at rest, or had an abnormal electrocardiogram prior to exercise. Similar to prior assessments at the baseline, Y7, and Y20 CARDIA exams, participants underwent a symptom-limited GXT using a modified Balke protocol with physician supervision^20^ at the Y35 follow-up exam to measure CRF.^13^ The modified Balke protocol consisted of a 2 min warm-up (2.0 mph, 0% grade) followed by up to nine 2 min stages (up to 18 minutes total) of progressively increasing difficulty, beginning at a workload of 4.1 METs and ending at 19.0 METs at stage nine, and a 3 min recovery (2.0 mph, 0% grade).^13^ The first stage in the protocol began at a treadmill speed of 3.0 mph and 2% grade, stages 2 through 6 maintained 3.4 mph with 6% grade for the stage 2 and then increased by 4% in each stage up to stage 6, 4.2 mph at 22% and 25% grade for stages 7 and 8, respectively, followed 5.6 mph at 25% grade for stage 9.

Heart rate, blood pressure, and a 12-lead ECG were obtained on each participant at rest (5 min), and heart rate, blood pressure, 12-lead ECG, and pulse oximetry were obtained at the end of each stage, at maximum exercise, and every minute for 3 min post-exercise. Respiratory exchange ratio (RER) and ratings of perceived exertion (RPE; 6-20 scale) were recorded near the end of each stage and at maximal exercise.^21^ Oxygen consumption was measured to obtain VO_2_max using an integrated metabolic measurement system (Ultima CardiO2, MGC Diagnostics, Saint Paul, Minnesota, United States). In addition to VO_2_max, total treadmill GXTd was recorded in total minutes and seconds and subsequently calculated as total seconds.

### Six-minute walk test

The 6MWT was conducted as part of the CARDIA Function ancillary study which included a battery of five physical performance measures and was administered by trained research staff who followed a standardized protocol and script. Participants were instructed to walk as fast as safely possible to cover as much distance during the 6 min period on an indoor hallway course approximately 30 feet in length. Participants were provided with updates on time remaining every minute (eg, 1 min down, 5 to go) and at the last 30 s. At the halfway point (ie, 3 min), participants were provided with verbal encouragement. The total distance covered during the period was recorded to the nearest meter.

### Comparison to existing VO_2_max prediction equations

Two existing prediction equations, the Burr equation^22^ and FRIEND equation,^23^ were used for comparison with the newly developed CARDIA-derived prediction equation. The Burr equation, developed in a sample of working-aged adults (aged 20-60 years), is frequently used for predicting VO_2_max from 6MWT distance. In addition to 6MWT distance, the equation incorporates body weight, sex, resting heart rate, and age as predictors of VO_2_max. The FRIEND equation, by contrast, is a non-exercise VO_2_max equation using age, sex, and body weight as predictor variables. The FRIEND equation was derived from the FRIEND registry, a diverse sample of healthy US men and women. These equations were selected because they are widely used in both clinical and research settings, represent different approaches to VO_2_max estimation (exercise-based vs non-exercise), and have been previously validated. We aimed to examine whether our newly developed equations would offer better or worse performance in predicting VO_2_max.

### Statistical analysis

For the current analyses, inclusion criteria included achieving at least one of the following indicators of a maximal effort during the GXT: exercise to volitional exhaustion, max heart rate ≥ 85% of age-predicted maximal heart rate using the Tanaka formula,^24^ and/or RER ≥ 1.10. Participant characteristics are reported, overall and stratified by sex, as means and standard deviations (SD) for continuous variables and proportions for categorical variables. Student’s *t* tests or Chi Square (*χ*^2^) tests were used to examine differences in participant characteristics by sex. The sample was stratified by sex, and stratified samples were randomly split into training sets (2/3 within strata) for model derivation and test sets (1/3 within strata) for validation. No additional cross-validation procedures were conducted because model performance was evaluated in this independent hold-out test set. Candidate predictors were selected a priori based on prior literature, clinical relevance, and practical availability, and included 6MWT distance (m), age (years), BMI (kg/m^2^), physical activity status, resting heart rate (bpm), blood pressure category, education, and smoking status. Bidirectional stepwise selection was then applied within the sex-specific training datasets to identify the final multivariable linear regression models for VO_2_max and GXTd. All model selection steps were confined to the training sets; final model performance was evaluated in the independent test sets. The models’ accuracies were evaluated in the test sets by comparing the measured and predicted VO_2_max (CARDIA, Burr, and FRIEND) and GXTd values using Student’s pairwise *t*-test and Pearson correlation coefficients (*r*). Additionally, Bland−Altman plots were generated to assess the agreement between measured and predicted values. All analyses were conducted in R (Version 4.3.2). Statistical significance was determined using an alpha level of 0.05.

## Results

The study sample comprised 564 participants, including 58.2% women and 40.4% Black individuals, with a mean age of 61.5 years (SD = 3.6, range 53-68) and a mean BMI of 28.6 kg/m^2^ (SD = 5.7). Participant characteristics of the overall sample and by sex are presented in Table 1. Men demonstrated higher height and weight, a higher proportion meeting physical activity guidelines, greater CRF (both VO_2_max and GXTd) and better 6MWT performance compared to women. Participants included in the analytical sample (consented to GXT, were medically eligible, and met inclusion criteria) were more likely to self-identify as White, have a lower BMI, have higher educational attainment, were more likely to meet physical activity guidelines, and have never smoked compared to the remaining Y35 CARDIA participants not included in the analyses (ie, did not complete both GXT and 6MWT) (Table S1).

**Table 1:** Participant characteristics, overall, and by sex.

	Total	Female	Male	*p*-value
Characteristics	*N* = 564	*N* = 328	*N* = 236	
**Testing center**				.91
Birmingham	138 (24.5%)	79 (24.1%)	59 (25.0%)	
Chicago	89 (15.8%)	53 (16.2%)	36 (15.3%)	
Minnesota	127 (22.5%)	71 (21.6%)	56 (23.7%)	
Oakland	210 (37.2%)	125 (38.1%)	85 (36.0%)	
**Race**				.006
Black	228 (40.4%)	149 (45.4%)	79 (33.5%)	
White	336 (59.6%)	179 (54.6%)	157 (66.5%)	
**Age (years)**	61.5 (3.6)	61.4 (3.7)	61.7 (3.5)	.30
**Height (cm)**	169.6 (9.3)	164.1 (6.7)	177.3 (6.7)	<.001
**Weight (kg)**	82.4 (18.1)	77.2 (17.9)	89.7 (15.8)	<.001
**Body mass index (kg/m^2^)**	28.6 (5.7)	28.6 (6.4)	28.5 (4.7)	.80
Healthy weight	163 (28.9%)	105 (32.0%)	58 (24.6%)	
Overweight	194 (34.4%)	103 (31.4%)	91 (38.6%)	
Obesity	207 (36.7%)	120 (36.6%)	87 (36.9%)	
**Education**				.21
Associate degree or more	383 (76.9%)	234 (79.1%)	149 (73.8%)	
High school or less	115 (23.1%)	62 (20.9%)	53 (26.2%)	
**Smoking status**				.43
Current	36 (6.5%)	17 (5.3%)	19 (8.1%)	
Former	115 (20.8%)	67 (21.0%)	48 (20.4%)	
Never	403 (72.7%)	235 (73.7%)	168 (71.5%)	
**Meeting physical activity guidelines**				.005
Yes	279 (50.0%)	145 (44.8%)	134 (57.3%)	
No	279 (50.0%)	179 (55.2%)	100 (42.7%)	
**Resting HR (bpm)**	65.1 (10.2)	65.6 (9.7)	64.4 (10.8)	.17
**Resting SBP (mmHg)**	121.3 (15.0)	121.0 (16.3)	121.8 (13.0)	.50
**Resting DBP (mmHg)**	72.4 (9.9)	72.2 (10.3)	72.6 (9.3)	.63
**GXT duration (s)**	329.7 (144.1)	281.5 (125.2)	396.4 (142.3)	<.001
**VO_2_max (mL/kg/min)**	26.0 (7.3)	24.0 (6.7)	28.9 (7.3)	<.001
**6MWT distance (m)**	477.4 (102.8)	467.7 (95.1)	490.8 (111.4)	.010

Each column reports mean (SD) or *N* (%). Student’s *t* and *χ*^2^-tests were used to examine potential sex differences. Not meeting or meeting physical activity guidelines based on a threshold of <300 or ≥300 exercise units. BMI categories: healthy weight: <25 kg/m^2^, overweight: 25.0-29.9 kg/m^2^, obesity ≥ 30 kg/m^2^.Abbreviations: 6MWT, six-minute walk test; DBP, diastolic blood pressure; GXT, graded exercise test; HR, heart rate; s, seconds; SBP, systolic blood pressure; VO_2_, maximal oxygen consumption.

### Predicting VO_2_max

The newly developed CARDIA sex-specific VO_2_max prediction equations following stepwise model selection are presented in Table 2. In both men and women, the final model included 6MWT distance, age, BMI, meeting or not meeting physical activity guidelines, resting heart rate, and smoking status as significant predictors of VO_2_max, resulting in adjusted *R*^2^ values of 0.53 and 0.57, respectively. Table 3 presents validation metrics for each equation, including mean predicted VO_2_max, mean difference from measured values with 95% confidence intervals, *p*-values, Pearson correlation coefficients (*r*), and standard error of the estimate (SEE) for men and women. Measured and predicted VO_2_max values based in their respective sex-specific equations are presented in Table 3. The differences between measured and predicted VO_2_max were close to 0 in both men and women (*p* = .63 and .20, respectively), with strong Pearson correlations between measured and predicted values in both men and women (*r* = 0.73 and 0.78, respectively).

**Table 2:** Newly developed cardiorespiratory fitness equations.

**VO_2_max (mL/kg/min)**	
Men	VO_2_ = 64.920 + (0.018 × 6MWT)−(0.344 × age)−(0.781 × BMI) + (2.934 × PA)−(0.099 × RestingHR)−(3.952 × Smoking)
Women	VO_2_ = 37.633 + (0.012 × 6MWT)−(0.044 × age)−(0.600 × BMI) + (2.209 × PA)−(0.044 × RestingHR)−(2.692 × Smoking)
**GXT duration (s)**	
Men	GXTd = 1110.422 + (0.393 × 6MWT)−(8.577 × age)−(12.465 × BMI) + (54.362 × PA)−(2.048 × RestingHR)−(83.698 × Smoking)
Women	GXTd = 667.671 + (0.373 × 6MWT)−(4.204 × age)−(9.275 × BMI) + (46.503 × PA)−(2.090 × RestingHR)−(82.922 × Smoking)

Inputs: 6MWT (meters); age (years); BMI (kg/m^2^), PA (0 = not meeting guidelines, 1 = meeting guidelines), RestingHR (bpm); smoking (0 = former or never smoker; 1 = current smoker).

Abbreviations: 6MWT, 6-minute walk test; BMI, body mass index; GXT, graded exercise test; PA, physical activity; RestingHR, resting heart rate; VO_2_, maximal oxygen consumption.

**Table 3:** Validation of newly developed cardiorespiratory fitness prediction equations.

Equation	Mean ± SD	Mean difference (95% CI)	*p*-value	*r*	SEE
**VO_2_max (mL/kg/min)**					
*Men*					
Measured	29.25 ± 6.99				
CARDIA Equation	29.52 ± 6.05	0.27 (−0.83, 1.37)	.627	0.73	4.81
Burr equation	32.38 ± 6.95	3.13 (1.81, 4.45)	<.001	0.65	6.56
FRIEND Equation	30.52 ± 4.80	1.27 (−0.03, 2.58)	.056	0.58	5.85
*Women*					
Measured	24.21 ± 7.22				
CARDIA Equation	23.64 ± 5.47	−0.57 (−1.44, 0.30)	.196	0.78	4.52
Burr equation	28.04 ± 6.80	3.83 (2.84, 4.82)	<.001	0.68	6.98
FRIEND equation	20.29 ± 5.18	−3.92 (−4.92, −2.92)	<.001	0.64	6.58
**GXT duration (s)**					
*Men*					
Measured	408.17 ± 148.96				
CARDIA equation	407.22 ± 113.44	−0.95 (−24.86, 22.96)	.937	0.71	104.67
*Women*					
Measured	291.75 ± 135.66				
CARDIA equation	280.14 ± 103.19	−11.61 (−29.76, 6.54)	.208	0.72	94.07

Abbreviations: Burr, Burr equation; CARDIA, CARDIA equation; FRIEND, FRIEND Registry Equation; GXT, graded exercise test; *r*, Pearson correlation; SEE, standard error of the estimate; VO_2_, maximal oxygen consumption.

**Figure 1:**
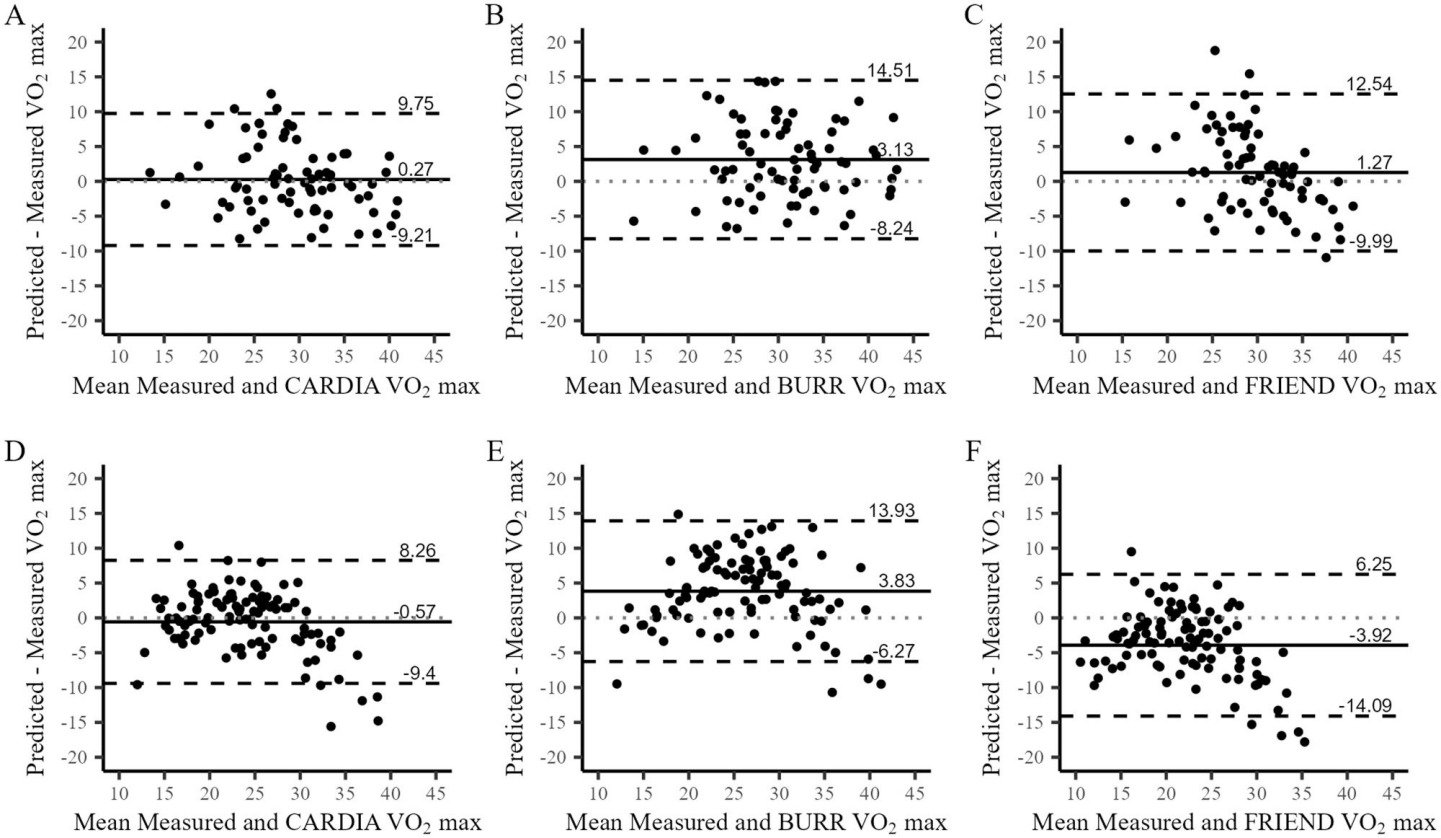
Bland−Altman Plots for predicted versus measured VO_2_max for men and women based on the CARDIA, Burr, and FRIEND equations. Solid center line represents mean bias. Dashed outer lines represent upper and lower limits of agreement (±1.96SD). Plots A, B, and C represent men; Plots D, E, and F represent women. BURR, Burr equation; CARDIA, CARDIA equation; FRIEND, FRIEND Registry Equation; VO_2_, maximal oxygen consumption.

The Burr equation, derived from a younger and less diverse cohort, produced VO_2_max estimates that overestimated measured values in this sample of middle-aged to older adult CARDIA participants by 3.13 mL/kg/min in men and 3.83 mL/kg/min in women, on average. In men and women, the Burr equation showed correlations with measured VO_2_max of *r* = 0.65 and 0.68, respectively, and SEE of 6.56 and 6.98 mL/kg/min. The FRIEND equation yielded VO_2_max predictions that were closer to measured values than the Burr equation but still demonstrated mean differences of 1.27 mL/kg/min for men and −3.92 mL/kg/min for women. Correlations with measured VO_2_max were *r* = 0.58 in men and *r* = 0.64 in women, and SEE values were 5.85 and 6.58 mL/kg/min, respectively. Bland−Altman plots comparing measured and predicted VO_2_max values for the newly developed CARDIA equation, Burr equation, and FRIEND equation are presented in Figure 1.

### Predicting GXTd

**Figure 2:**
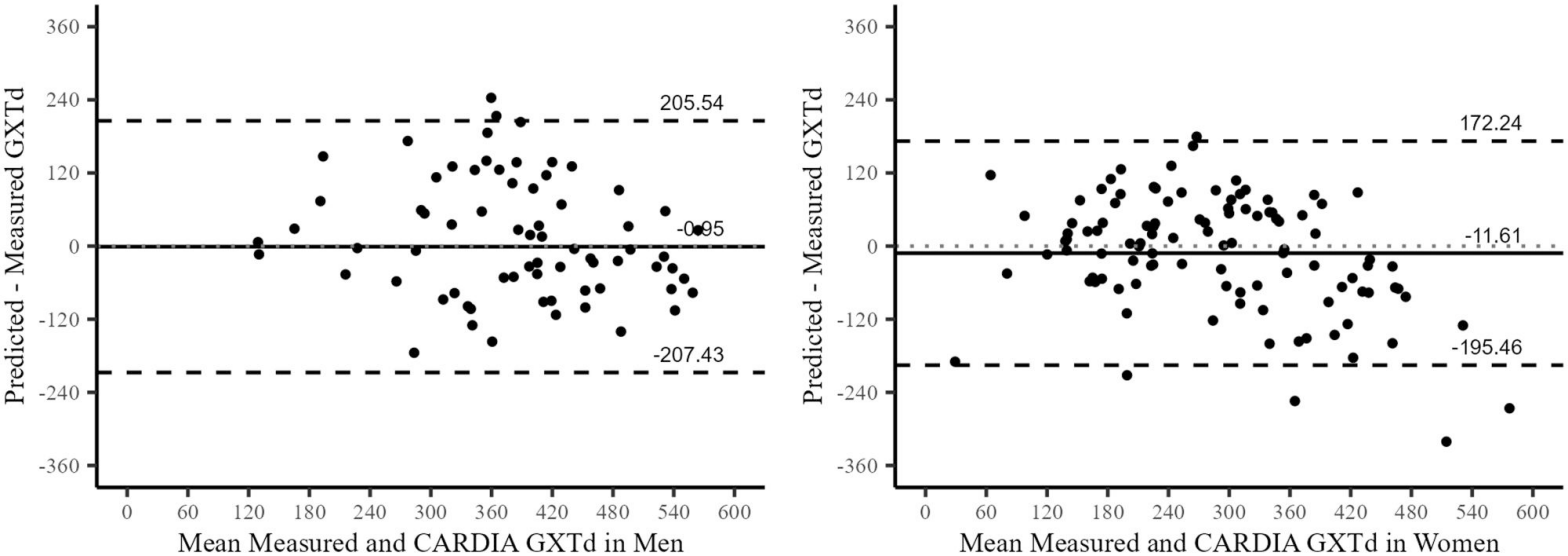
Bland−Altman Plots for predicted versus measured GXTd for men and women based on the CARDIA equation. Solid center line represents mean bias. Dashed outer lines represent upper and lower limits of agreement (±1.96 SD). CARDIA, CARDIA equation; GXTd, graded exercise test equation.

6MWT distance, age, BMI, physical activity status, resting HR, and smoking status were significant predictors of GXTd in both men and women (Table 2). Presented in Table 2, the newly developed CARDIA prediction equations resulted in adjusted *R*^2^ values of 0.59 and 0.62 in men and women, respectively. The differences between measured and predicted GXTd were close to 0 in both men and women (*p* = .94 and .21, respectively), with strong Pearson correlations between measured and predicted values in both men and women (*r* = 0.71 and 0.72, respectively) (Table 3). In contrast to VO_2_max, GXTd was underestimated in both men and women (mean differences: −0.95 s and −11.61 s, respectively). Bias and limits of agreement for GXTd measured and predicted values are presented in Bland Altman plots in Figure 2.

## Discussion

The results of this study underscore the utility of the 6MWT as a practical and accurate measure to include in models estimating CRF, both VO_2_max and GXTd, in large-scale, population-based studies. Our findings build upon previous research demonstrating the validity of 6MWT distance as a predictor of VO_2_max, with further development of sex-specific prediction equations for both VO_2_max and GXTd in a population-based sample of middle-aged to older adults. The strong correlation between predicted and measured values for both VO_2_max and GXTd, along with the lack of significant differences, indicates that the prediction models are robust. The inclusion of age, BMI, meeting or not meeting physical activity guidelines, resting heart rate, and smoking status as predictors in addition to 6MWT distance in our models provide additional, simple to assess, participant characteristic information aiding in the accuracy of CRF prediction.

The new VO_2_max CARDIA prediction equations for men and women explained 53% and 57% of the variance in VO_2_max, respectively, with strong correlations between measured and predicted VO_2_max values (*r* = 0.73 and 0.78). Although small biases were observed, with VO_2_max being slightly overestimated in men and underestimated in women, the mean differences were not statistically significant, indicating that the equations performed well across sex-specific groups. For context, the Burr equation,^22^ selected due to its widespread use in estimating VO_2_max from 6MWT distance, overestimated VO_2_max in this sample in both men and women. Correlations between predicted and measured VO_2_max were *r* = 0.65 for men and *r* = 0.68 for women. This overestimation is likely attributable to differences in the samples used for equation development; the Burr equation was derived from a sample of healthy, working-aged individuals with higher CRF levels than those in our study. The FRIEND equation,^23^ developed using a diverse sample of healthy US men and women, also showed mean differences from measured VO_2_max values, tending to overestimate VO_2_max in men and underestimate it in women, with correlations of *r* = 0.58 and *r* = 0.64. Together, these observations highlight how VO_2_max prediction equations derived in distinct populations yield different levels of agreement when applied to a middle-aged to older adult cohort.

The newly developed GXTd prediction equations also demonstrated robust performance with *R*^2^ values of 0.59 and 0.62 in men and women, respectively. Measured and predicted GXTd values were strongly correlated (*r* = 0.71 and *r* = 0.72, respectively) and showed no significant differences (*p* = .94 and *p* = .21, respectively). Highlighting the utility of 6MWT distance for estimating GXTd in large-scale studies where time to exhaustion is used as a proxy for CRF. By accurately estimating GXTd, our equations expand the application of the 6MWT, offering an additional, practical metric for CRF assessment that aligns with a historical treadmill protocol in CARDIA.^13^ Moreover, these equations provide a valuable tool for longitudinal cohort studies, such as CARDIA, where treadmill-based tests were routinely performed in earlier study phases but may no longer be feasible as cohort participants age. The ability to harmonize historical treadmill data with current 6MWT performance ensures continuity in CRF assessment and supports the exploration of CRF trends and their associations with long-term health outcomes.

Despite the strengths of this study, several limitations should be acknowledged. First, while the newly developed VO_2_max and GXTd prediction equations demonstrated robust predictive performance, the explained variance (*R*^2^) remains moderate, indicating that additional predictors could improve accuracy of the estimates. Including variables such as objectively measured physical activity or body composition measures might improve model performance but would increase complexity and resource demands, potentially reducing the equations’ practical utility. Second, these equations were developed using data from the CARDIA cohort including the CARDIA-modified Balke treadmill protocol and 6MWT. As such, their performance may not generalize to other populations or to studies employing different exercise testing protocols, particularly for GXTd predictions. Furthermore, participants had to enroll in the GXT ancillary study and meet ancillary specific inclusion criteria which introduces a potential selection bias, as individuals willing to participate in a GXT may possess a certain level of fitness and physical function, potentially influencing the study outcomes.^25^ Additionally, The CARDIA Y35 in-person exam occurred during COVID-19, possibly limiting further limiting participation. Finally, our results may not be generalizable to other race and ethnic groups (eg, Asian adults) or age groups, as they were not represented in CARDIA. Future research should validate these equations across diverse cohorts and protocols to ensure broader applicability.

## Conclusions

The development of sex-specific prediction equations for VO_2_max and GXTd from 6MWT performance represents a significant advancement in the field of CRF assessment. These equations are particularly valuable for large-scale epidemiological studies where traditional GXT may not be feasible.

## Supplementary Material

glag040_Supplementary_Data

## Data Availability

CARDIA data are available upon reasonable request from the CARDIA Coordinating Center. CARDIA investigators are eager to collaborate with investigators interested in using CARDIA data. Please see the CARDIA website (https://sites.uab.edu/cardia/for-researchers/publications/) for publication policies and for a list of CARDIA Representatives. CARDIA data are also publicly available on the NIH-supported BioLINCC and dbGaP platforms.
