# Supplementary material for: Development and validation of cardiorespiratory fitness prediction equations from 6-minute walk test: the Coronary Artery Risk Development in Adults (CARDIA) study
Source: J Gerontol A Biol Sci Med Sci. 2026 Feb 14;81(4):glag040. doi: 10.1093/gerona/glag040 (PMC12944221; doi:10.1093/gerona/glag040)
Supplement: glag040_Supplementary_Data [file glag040_supplementary_data.pdf]

Supplementary Material for 'Development and Validation of Cardiorespiratory Fitness Prediction Equations from 6-Minute Walk Test in the CARDIA Study'

Contents

Supplemental Table 1. Differences in participant characteristics of the analytic sample versus the Y35 CARDIA participants not included in the analyses.

Supplemental Table 1. Differences in participant characteristics of the analytic sample versus the Y35 CARDIA participants not included in the analyses.

	**Analytic Sample**	**Participants Attending the (in-person) Year 35 exam**	**p-value**
Characteristics	N = 564	N = 1,685	
Testing Center (%)			<0.001
Birmingham	138 (24.5%)	431 (25.6%)	
Chicago	89 (15.8%)	412 (24.5%)	
Minnesota	127 (22.5%)	469 (27.8%)	
Oakland	210 (37.2%)	373 (22.1%)	
Race (%)			0.027
Black	228 (40.4%)	772 (45.8%)	
White	336 (59.6%)	913 (54.2%)	
Sex (%)			0.50
Female	328 (58.2%)	952 (56.5%)	
Male	236 (41.8%)	733 (43.5%)	
Age (yrs)	61.5 (3.6)	61.3 (3.6)	0.22
Height (cm)	169.6 (9.3)	169.1 (9.4)	0.30
Weight (kg)	82.4 (18.1)	88.8 (22.3)	<0.001
BMI (kg/m^2)	28.6 (5.7)	31.0 (7.5)	<0.001
Healthy weight (%)	163 (28.9%)	337 (20.2%)	
Overweight (%)	194 (34.4%)	547 (32.8%)	
Obesity (%)	207 (36.7%)	783 (47.0%)	
Education (%)			0.002
Associate degree or more	383 (76.9%)	925 (69.4%)	
High school or less	115 (23.1%)	408 (30.6%)	
Smoking Status (%)			0.006
Current	36 (6.5%)	168 (10.4%)	
Never or former	518 (93.5%)	1,451 (89.6%)	
Meeting physical activity guidelines (%)			<0.001
Yes	279 (50.0%)	636 (38.4%)	
No	279 (50.0%)	1,019 (61.6%)	

Note: Each column reports mean (SD) or N (%). Student's t-tests and chi-squared tests were used to examine potential differences, as appropriate.
BMI categories: healthy weight: < 25 kg/m^2, overweight: 25.0-29.9 kg/m^2, obesity ≥ 30 kg/m^2. Not meeting or meeting physical activity guidelines based on a threshold of <300 or ≥300 exercise units.
